# Panels of HIV-1 Subtype C Env Reference Strains for Standardized Neutralization Assessments

**DOI:** 10.1128/JVI.00991-17

**Published:** 2017-09-12

**Authors:** Peter Hraber, Cecilia Rademeyer, Carolyn Williamson, Michael S. Seaman, Raphael Gottardo, Haili Tang, Kelli Greene, Hongmei Gao, Celia LaBranche, John R. Mascola, Lynn Morris, David C. Montefiori, Bette Korber

**Affiliations:** a Theoretical Biology and Biophysics, Los Alamos National Laboratory, Los Alamos, New Mexico, USA; b Division of Medical Virology & Institute of Infectious Diseases and Molecular Medicine, University of Cape Town and NHLS, Cape Town, South Africa; c Center for Virology and Vaccine Research, Beth Israel Deaconess Medical Center, Harvard Medical School, Boston, Massachusetts, USA; d Vaccine and Infectious Disease Division, Fred Hutchinson Cancer Research Center, Seattle, Washington, USA; e Department of Surgery, Duke University Medical Center, Durham, North Carolina, USA; f Vaccine Research Center, National Institute of Allergy and Infectious Diseases, National Institutes of Health, Bethesda, Maryland, USA; g National Institute for Communicable Diseases, Johannesburg, South Africa; h New Mexico Consortium, Los Alamos, New Mexico, USA; Ulm University Medical Center

**Keywords:** assay standardization, clinical trials, human immunodeficiency virus, immunoserology, neutralizing antibodies, vaccines

## Abstract

In the search for effective immunologic interventions to prevent and treat HIV-1 infection, standardized reference reagents are a cost-effective way to maintain robustness and reproducibility among immunological assays. To support planned and ongoing studies where clade C predominates, here we describe three virus panels, chosen from 200 well-characterized clade C envelope (Env)-pseudotyped viruses from early infection. All 200 Envs were expressed as a single round of replication pseudoviruses and were tested to quantify neutralization titers by 16 broadly neutralizing antibodies (bnAbs) and sera from 30 subjects with chronic clade C infections. We selected large panels of 50 and 100 Envs either to characterize cross-reactive breadth for sera identified as having potent neutralization activity based on initial screening or to evaluate neutralization magnitude-breadth distributions of newly isolated antibodies. We identified these panels by downselection after hierarchical clustering of bnAb neutralization titers. The resulting panels represent the diversity of neutralization profiles throughout the range of virus sensitivities identified in the original panel of 200 viruses. A small 12-Env panel was chosen to screen sera from vaccine trials or natural-infection studies for neutralization responses. We considered panels selected by previously described methods but favored a computationally informed method that enabled selection of viruses representing diverse neutralization sensitivity patterns, given that we do not *a priori* know what the neutralization-response profile of vaccine sera will be relative to that of sera from infected individuals. The resulting 12-Env panel complements existing panels. Use of standardized panels enables direct comparisons of data from different trials and study sites testing HIV-1 clade C-specific products.

**IMPORTANCE** HIV-1 group M includes nine clades and many recombinants. Clade C is the most common lineage, responsible for roughly half of current HIV-1 infections, and is a focus for vaccine design and testing. Standard reference reagents, particularly virus panels to study neutralization by antibodies, are crucial for developing cost-effective and yet rigorous and reproducible assays against diverse examples of this variable virus. We developed clade C-specific panels for use as standardized reagents to monitor complex polyclonal sera for neutralization activity and to characterize the potency and breadth of cross-reactive neutralization by monoclonal antibodies, whether engineered or isolated from infected individuals. We chose from 200 southern African, clade C envelope-pseudotyped viruses with neutralization titers against 16 broadly neutralizing antibodies and 30 sera from chronic clade C infections. We selected panels to represent the diversity of bnAb neutralization profiles and Env neutralization sensitivities. Use of standard virus panels can facilitate comparison of results across studies and sites.

## INTRODUCTION

The quest to induce and understand protective immune responses to HIV-1 elicited by vaccination remains a high priority. Passive administration of broadly neutralizing antibodies (bnAbs) is also being evaluated for its ability both to prevent and to treat HIV-1 infection. Use of standardized reference reagents facilitates comparisons of results from different cohorts or trials (1). The demand for reagents that reflect global diversity of HIV-1 is offset by the overwhelming regional burden of specific forms of the virus. This regional burden is acutely clear for clade C viruses in southern Africa.

Clade C is far more common than any other HIV-1 lineage. For the period 2004 to 2007, nearly half (48%) of all HIV-1 infections were clade C, representing an estimated 15.8 million people (2). It is the dominant clade in southern Africa and India, and circulating recombinants that include C clade Env regions are very common in China (3). Although those prevalence estimates were current a decade ago, as of March 2017, sequences collected in the HIV database (http://hiv.lanl.gov/components/sequence/HIV/geo/geo.comp) indicated that C clade predominated in South Africa (98% of 32,826 sequences were C clade) and India (95% of 13,475 sequences were C clade) and that C clade or BC recombinants were present in roughly half of 30,188 sequences from China. Furthermore, multiple lines of evidence suggest that clade C is more transmissible (4–6) and may have greater replicative fitness (7, 8) than other subtypes, so its prevalence is unlikely to have decreased in the past 10 years. The next most abundant nonrecombinant forms are clade A (12%) and clade B (11%), present in 3.9 and 3.7 million individuals, respectively. Recombination is also very common, with circulating recombinant forms (CRFs) and unique, noncirculating recombinants (URFs) together constituting 20% (6.7 million) of the infections (2).

Here we describe development of standard clade C virus panels for two anticipated uses. Sets of 50 and 100 Envs are intended to enable detailed characterization of magnitude-breadth distributions for neutralizing antibodies and sera. A smaller, more manageable set of 12 Envs is intended for use in screening newly isolated antibodies or sera from vaccinees. The 12-Env C clade panel was selected to include informative examples of neutralization specificities that arise during the course of natural C clade infections. Envs for these neutralization panels were chosen from a set of 200 well-characterized clade C Envs which we recently described elsewhere (9). The Envs represent the HIV-1 genetic and antigenic diversity of members of clade C in southern Africa and do not include other geographic regions, such as India (9). A primary goal was to enable detection of neutralization responses in the new HVTN 702 vaccine efficacy trial that has recently begun in South Africa (10), wherein immune responses to a clade C vaccine will be monitored for their capacity to prevent infection in a clade C epidemic (11).

In related work, we recently described development of a 12-virus global panel that captures average neutralization responses corresponding to the M (main) group diversity, including common subtypes and CRFs (12). Both the global panel and the virus panels that we developed in the present study are intended to provide standardized reagents to investigators working to characterize adaptive immune responses to HIV. The panels developed here differ from the global reference panel in that the Envs are all from clade C, whereas the 12-virus global panel contains more genetic diversity as a consequence of inclusion of clades A, B, C, and G plus the recombinants CRF01 and CRF07. Also, a main selection criterion for the global panel was the ability to infer typical (median) serum potency. To that end, we identified nine viruses that satisfied the criterion optimally and then added three viruses deliberately chosen to include patterns of neutralization response diversity that were not otherwise included (12). In contrast, here we describe a clade C panel of 12 Envs intended to detect relatively weak or potentially clade-specific tier 2 neutralization responses. Vaccine sera that yielded any detectable response(s) could be identified for further evaluation. Ultimately, both the clade C and M group panels are intended for use in vaccine trials and in other settings.

## RESULTS

### Antibody neutralization.

**FIG 1:**
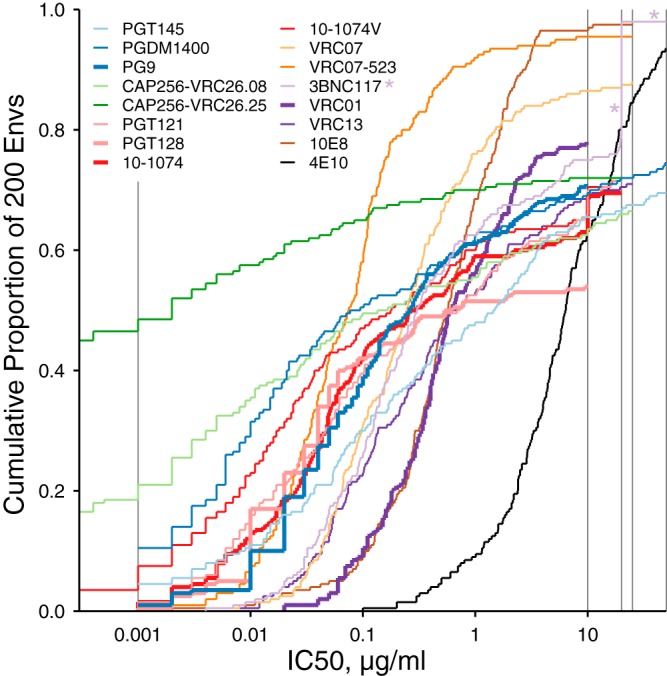
Cumulative distributions of IC_50_ neutralization titers from 16 bnAbs. Each line shows the proportion of 200 Envs, with IC_50_s given by the value along the *x* axis. Gray lines at the lower and upper ranges of IC_50_s indicate where censoring cutoffs differed among assays. Asterisks are intended to help locate the example of 3BNC117 censored at 20 and 50 μg/ml discussed in the text.

Neutralization titers are typically determined as point values (e.g., 50% inhibitory concentration [IC_50_] and IC_80_ values) to summarize distributions from a series of reagent concentrations. The antibody concentration ranges tested in neutralization assays often produce censored neutralization IC_50_ titers, where the range of concentrations does not yield 50% neutralization. Censored outcomes are represented as “>*x*,“ where *x* is the greatest concentration used, or “<*y*,” where *y* is the lowest concentration used. These cutoffs can differ across assays, generally due to practical constraints of limited serum or antibody availability. Such censoring is an issue for quantitative analysis, because standard practice would use a constant placeholder value for censored outcomes; e.g., an IC_50_ value above 50 (“>50”) is replaced with a value of 100. Censoring thresholds of 10, 20, 25, and 50 μg/ml were used for different bnAbs (Fig. 1), and it was sometimes necessary to use different thresholds for even a single bnAb, such as 3BNC117. Most of the IC_50_ titers corresponding to 3BNC117 were not censored (*n* = 158 Envs). However, the 3BNC117 values were reported as >20 μg/ml for 38 Envs, and the values were >50 μg/ml for 4 Envs. To standardize the comparisons and to compare different bnAbs against the 200-virus panel, we used a consistent censoring cutoff of 10 μg/ml across all assays, and IC_50_s below 0.01 μg/ml were censored at 0.01.

### Magnitude-breadth panels.

**FIG 2:**
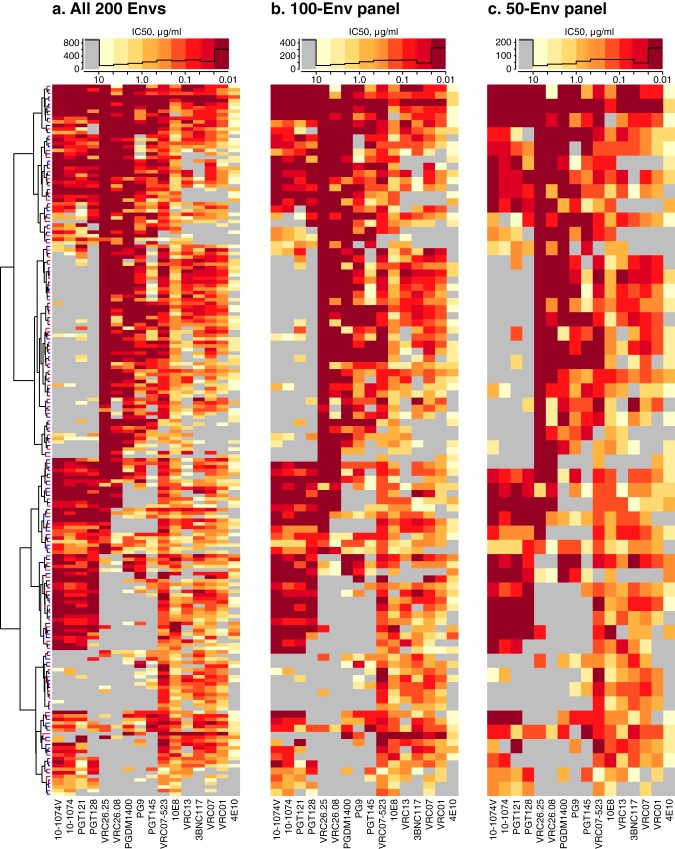
Heat maps of IC_50_ neutralization titers from assaying 200 clade C envelopes against 16 bnAbs. (a) Hierarchically clustered heat map of IC_50_ titers of 200 Envs against 16 bnAbs. The Env dendrogram is shown; the bnAb dendrogram is not shown. Leaf colors indicate 100 viruses included (red) or excluded (blue) by downselection. The histogram (black line) above the heat map summarizes the distribution of assay results, with histogram breakpoints at 10, 4.64, 2.15, 1.00, 0.464, 0.215, 0.10, 0.0464, 0.0215, and 0.01 μg/ml. Low IC_50_s were censored at 0.01 μg/ml to standardize censoring thresholds across bnAbs. (b) 100-Env panel, downselected from alternating rows, i.e., red branches on the dendrogram. (c) 50-Env panel downselected by alternating over 100 Envs.

The Envs downselected for magnitude-breadth characterization sampled the spectrum of bnAb reactivity patterns from the full set of 200 Envs (Fig. 2). Heat maps show IC_50_ titers for the full neutralization panel (Fig. 2a) and for downselected panels of 100 Envs (Fig. 2b) and 50 Envs (Fig. 2c). Histograms show similar IC_50_ distributions (combined for all 16 bnAbs) at the top of each panel.

**FIG 3:**
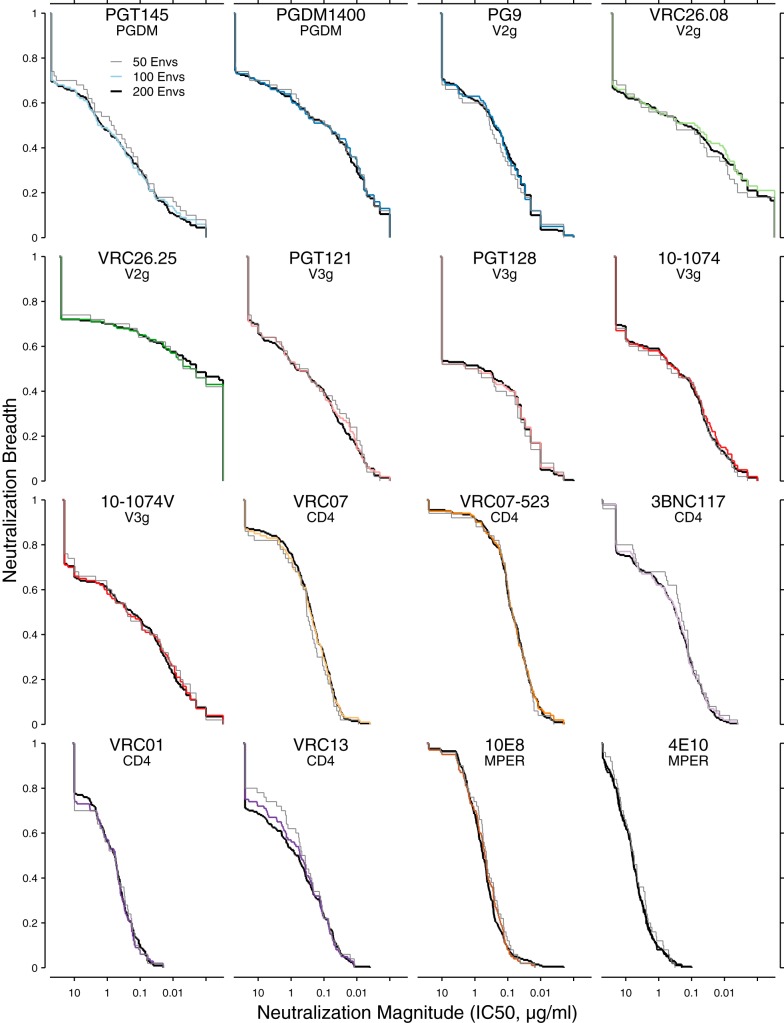
Magnitude-breadth curves (cumulative distribution functions) compare IC_50_s from 100- and 50-Env panels (colored and gray lines, respectively) with those from all 200 Envs (black lines). Axes are scaled the same across all panels. Specificities for each bnAb are also listed. The curves are continuous distribution functions which indicate the proportion of viruses with IC_50_ neutralization titers (expressed in micrograms per milliliter) no lower than the corresponding *x* axis value. Because the emphasis here is on comparisons within the same bnAb, the differences in censoring noted for Fig. 1 across different bnAbs are not relevant here. The differences are quantified in the supplemental material.

For each of the bnAbs, Fig. 3 compares neutralization magnitude-breadth distributions of the full panel of 200 clade C Envs with the distributions of the downselected panels. In most cases, the magnitude-breadth distributions show a high degree of overlap, which means that the downselected panels are a good representation of the properties of the full set. A slight shift toward greater neutralization sensitivity is apparent for some bnAbs, where distributions of selected Envs are biased toward IC_50_ values slightly lower than those seen with the excluded Envs. This small bias resulted from favoring more-sensitive viruses in choosing alternate rows in the heat map, i.e., by starting with the most sensitive virus rather than skipping it for the next most sensitive.

The concordance of the breadth-potency curves was very high and consistent across bnAbs for the sets of 100 and 50 Envs (see Table S1 in the supplemental material). Downsampling further to obtain a 12-Env panel increased the bias in favor of some bnAbs and against others and gave only a rough approximation of the full set of 200 Envs (see Fig. S1 in the supplemental material). Also, downsampling to 12 Envs greatly increased the area between the magnitude-breadth curves versus the area seen with the full set (Fig. S2), thus accounting for part of our rationale not to use downsampling to select a 12-Env panel. We instead considered other approaches.

### Serum screening panel (12 Envs).

**FIG 4:**
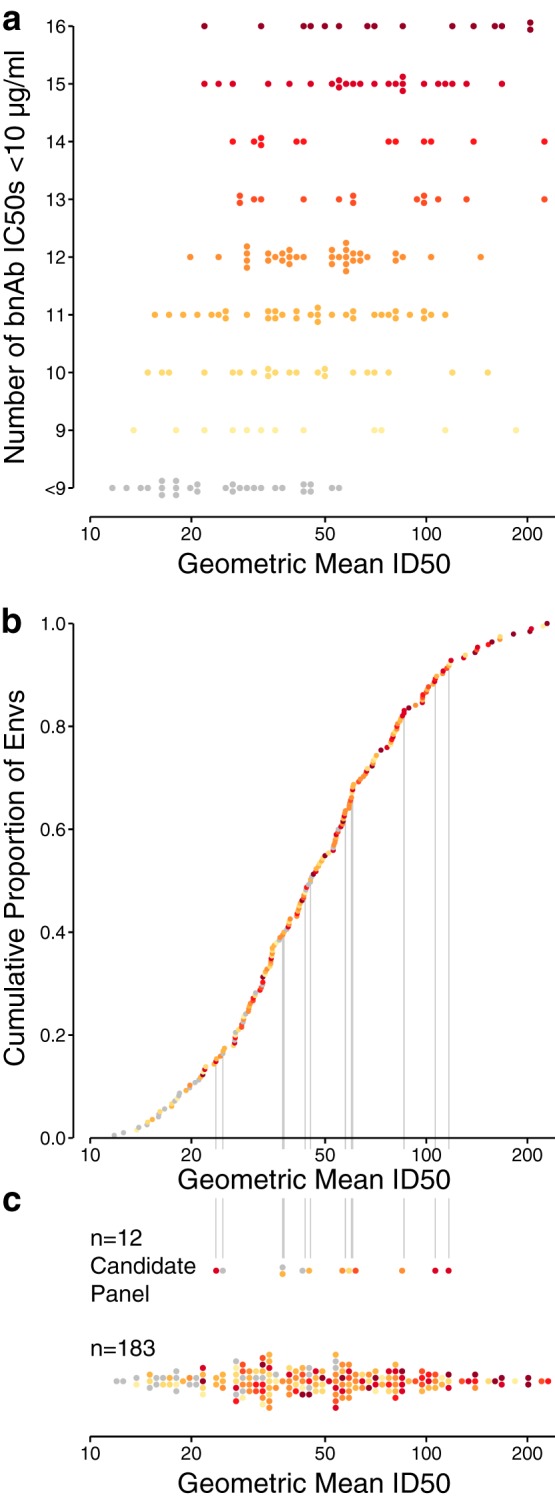
Comparison of Env sensitivity to neutralization by plasma samples and bnAbs. (a) Geometric mean plasma ID_50_ per Env, stratified by the number of bnAbs with IC_50_s below 10 μg/ml, from among the 16 tested. (b) Cumulative distribution of geometric mean ID_50_s from 30 chronic plasma samples. Vertical lines indicate the 12 selected Envs. (c) Distribution of geometric mean ID_50_s between 12-virus panel and nonpanel Envs. Five Envs with geometric mean ID_50_s above 250 μg/ml are not shown. Colors in panels b and c summarize the number of neutralizing bnAbs shown in panel a.

Figure 4a summarizes Env sensitivity to neutralization by plasma, calculated as geometric mean ID_50_ among 30 chronic plasma samples, together with the number of bnAbs that neutralized each Env. This coarse measure of sensitivity across all bnAbs was significantly associated with sensitivity to plasma (Kendall's τ, τ = 0.338, *P* = 3.34 × 10^−11^). We used this association to select Envs from principal-coordinate analysis (PCA) of bnAb neutralization data via computational guidance.

Informed by the results from testing each Env against multiple bnAbs, we sought to represent the diversity of different bnAb specificities, to reduce the risk of missing neutralization signal by overrepresenting the most common bnAb specificities. For this reason, we selected 12 Envs to represent a range of neutralization sensitivities to polyclonal plasma and monoclonal antibodies. Figure 4b shows the cumulative distribution of Env sensitivities to plasma. Env colors indicate the number of bnAbs with an IC_50_ value below 10 μg/ml from Fig. 4a. Where plasma and bnAb sensitivities are closely associated, the progression of Envs appears in an order consistent with the progression of rows in Fig. 4a. An overall trend is apparent for an association between serum and bnAb sensitivity, though small inconsistencies across Envs reflect wide variations in neutralization titers against sera and in the number of bnAbs to which each Env is sensitive.

**FIG 5:**
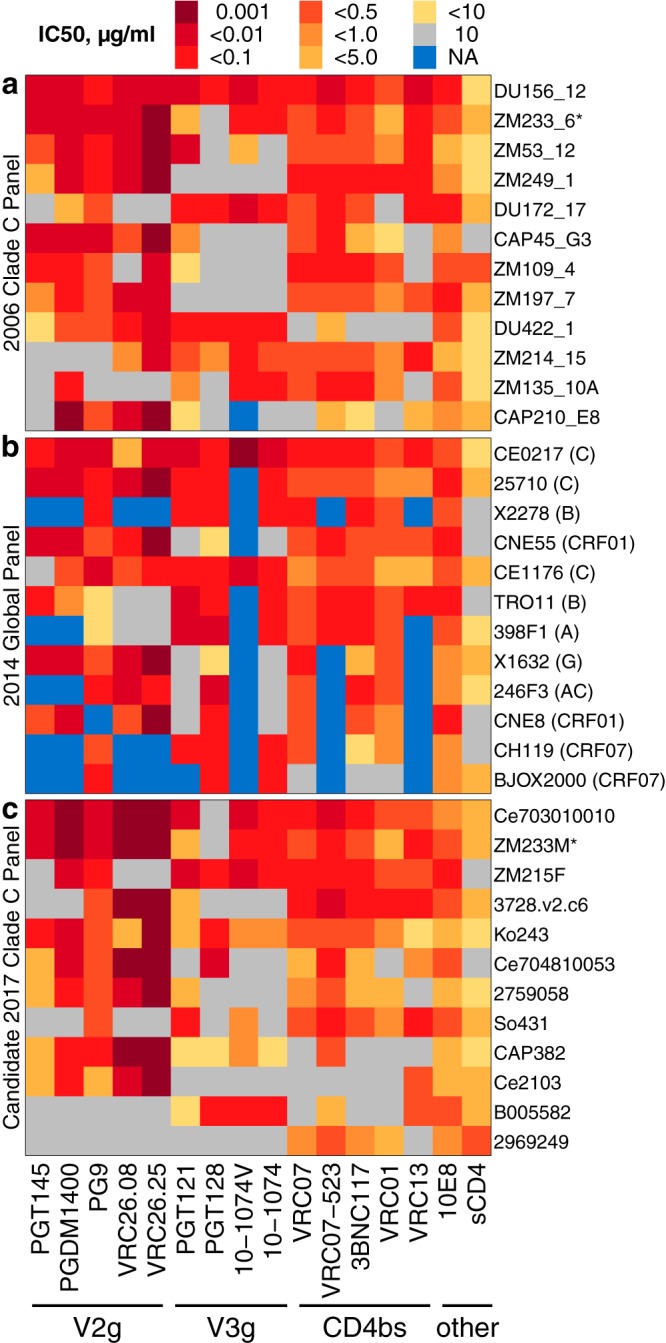
Comparison of neutralization IC_50_ titers between 12-Env panels. (a to c) Comparison of (a) clade C panel from 2006, (b) global panel (12), and (c) candidate clade C panel from the manuscript. As noted in the Fig. 1 legend, all assay results were censored above 10 and below 0.001 μg/ml to standardize dilution ranges across different experiments. NA, no data. Data for historical panels a and b were computed as geometric means from the CATNAP database, as detailed in the text. Env names in panels a and b are shortened per CATNAP, and panel c lists short names from Data Set S1.

Figure 4c compares plasma ID_50_ distributions between the Envs in the candidate panel and the remaining Envs. The candidate panel was intentionally chosen to avoid extremely high or low geometric mean ID_50_ titers among chronic plasma samples, both to reduce false negatives and to exclude tier 1 neutralization responses, which are readily obtained in induced form and do not correlate with immune protection (13, 14). We found no evidence that the geometric mean ID_50_s of the Envs in the selected panel (*n* = 12) and the remaining Envs (*n* = 183) were sampled from different distributions (two-sided, two-sample Kolmogorov Smirnov test, *P* = 0.53). The candidate panel Envs were neutralized by different numbers (Fig. 4) and subsets (Fig. 5) of bnAbs, rather than the Envs being sensitive to all the bnAbs studied, and we confirmed that multiple Envs that were well targeted by each major monoclonal antibody epitope specificity tested were included.

To simplify the diverse outcomes of Env sensitivity to neutralization by different antibodies and to facilitate the selection of 12 Envs that covered a range of distinctive neutralization profiles with respect to the 16 bnAbs tested, we used PCA, which flattens the neutralization data into orthogonal (minimally correlated) sets of linear combinations of bnAbs (Fig. S3). The first two principal components together explained about half (47.6%) of the variance in the bnAb IC_50_ data. Adding the third principal component accounted for 64.6% of the total variance. As detailed in the supplemental material, the first three principal components were strongly associated with combinations of CD4 binding-site (CD4bs), V2 glycan, and V3 glycan bnAb specificities.

**FIG 6:**
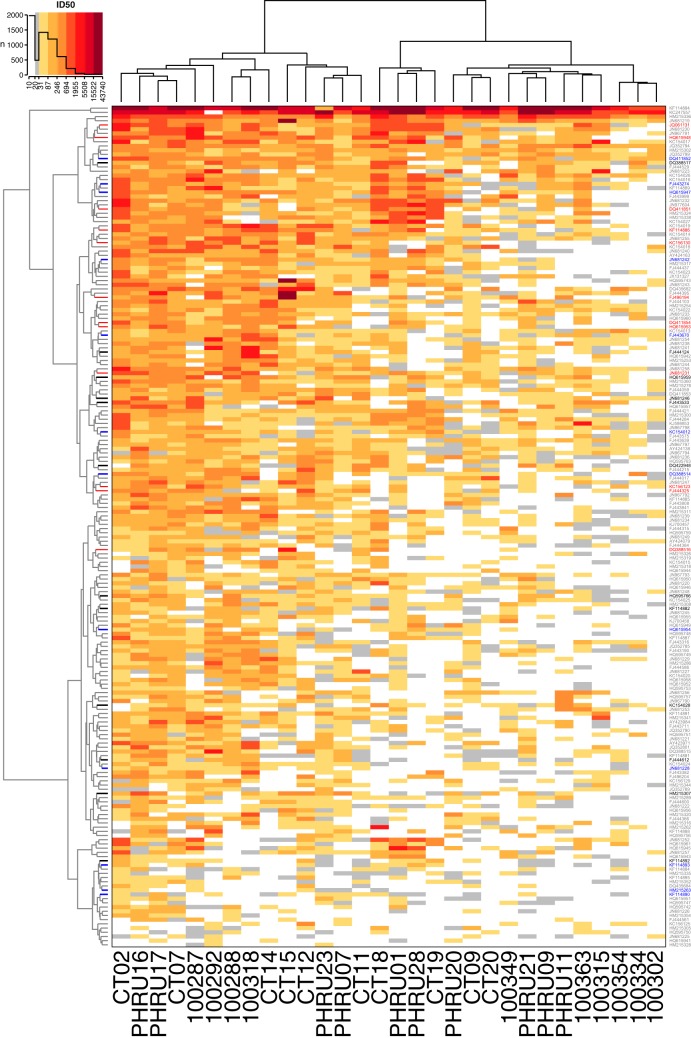
Hierarchically clustered dendrogram of 200 tier 2 envelopes with heat map of neutralization ID_50_s. The dendrogram was computed from squared Euclidean distance values using Ward's clustering method. Leaves (rows) were weighted by geometric mean neutralization titer for dendrogram layout. Colors indicate viruses selected for the candidate 12-Env panel (black). Panels defined by the automatic methods for lasso (red) and by *k*-medoids (blue) with *k* = 12 are also indicated. Other virus names are indicated in gray.

On the basis of comparisons of the alternative clustering methods, we favored the use of Ward's method (15) with squared Euclidean distances (ward.D2) for clustering. Ward's method was best able to cluster distinctive patches of serum and virus specificities within the broader gradient of plasma neutralization sensitivities. The resulting clustered heat map of serum neutralization ID_50_ titers (Fig. 6) is annotated to identify the candidate panel of 12 Envs. The panels identified automatically (lasso and *k*-medoids), as described in the supplemental material, are also shown for comparison. All three sets of Envs represent a range of average neutralization sensitivities, as reflected by their dispersal from the top to the bottom of the heat map, which corresponded to more-resistant and more-sensitive Envs, respectively. The candidate panel, chosen with computational guidance, covers a more limited range of sensitivities than the automatically chosen Envs. This was done intentionally to avoid both highly sensitive and very resistant viruses during the iterative procedure described above.

Other clustering methods can yield quite different outcomes, and the correlation coefficient between cophenetic distances (16) summarizes similarity among clusters obtained using alternative algorithms (Fig. S4).

Ordering ID_50_s by geometric mean titer reveals the continuum of neutralization responses (Fig. S5) that are characteristic of the polyclonal mixture of antibody potencies and/or specificities found in plasma samples (17). This continuum further emphasizes the benefit of using bnAb sensitivities, rather than plasma responses, for computationally guided panel selection, given that we do not know whether a range of antibody sensitivities or of various antibody potencies dominates the neutralization response of any plasma sample.

Fig. S6 summarizes serum neutralization responses among the 12-Env panels identified by 3 automated methods (downselection, lasso, and *k*-medoids) versus computationally guided selection. Because computationally guided selection avoided individual Envs that were sensitive to all bnAb specificities, the candidate panel does not merely reflect the continuum of neutralization responses, as do the panels identified by automated methods. Sensitive and informative detection of tier 2 neutralization responses, not modeling the full distribution of Env plasma sensitivities, is the main purpose intended for the candidate 12-Env panel.

Information about the 12 candidate Envs, including the geographic region and year sampled, is summarized in Table 1. Other information is tabulated to summarize genetic attributes of these sequences, including the glycosylation state (presence or absence of a potential N-linked glycosylation motif) at sites relevant to antibody binding susceptibility, hypervariable loop lengths and net charges, and the infection stage from which the virus was sampled.

**TABLE 1:** Properties of tier 2 Envs selected for candidate 12-virus panel, chosen with computational guidance

Accession no.^*a*^	Name^*b*^	Yr^*c*^	Country/province^*d*^	is TF^*e*^	Stage^*f*^	N332 or N334^*g*^	N293 or N295^*g*^	N130^*g*^	N156 or N160^*g*^	No. of V1V2 aa^*h*^	V1V2 charge^*i*^	V4 length	V4 charge	V5 length	V5 charge
FJ443533	Ce703010010	2006	MW	T	A1	N332	None	F	Both	28	2	4	−1	9	−4
DQ388517	ZM233 M	2002	ZM	NA	E	N332	N293	F	Both	19	2	4	0	7	−1
DQ422948	ZM215F	2002	ZM	NA	E	N332	None	T	Both	15	0	4	1	5	0
HM215307	3728	2004	TZ	F	A2	None	N295	F	Both	22	−2	9	1	6	0
KF114892	Ko243	2009	ZA/nw	T	E	N332	N295	F	Both	49	2	13	0	7	2
FJ444124	Ce704810053	2007	ZA/gp	T	A1	None	N295	T	Both	34	−2	9	3	4	0
HQ615959	2759058	2006	ZA/kz	T	A1	None	None	T	Both	23	−1	6	−1	10	−2
JN681246	So431	2007	ZA/gp	T	E	N332	None	F	Both	31	−1	8	0	7	−1
KC154028	CAP382	2010	ZA/kz	T	E	N332	None	F	Both	22	−5	5	1	11	−2
FJ444612	Ce2103	2005	MW	T	A1	N334	None	T	N156	34	−8	12	−1	6	0
KF114882	B005582	2007	BW	T	A2	N332	N295	F	Both	31	−3	7	0	8	−1
HQ595766	2969249	2007	ZA/kz	T	A2	N334	N295	F	Both	31	0	7	−1	8	−1

a A table with these data for all 200 Envs is available in the supplemental material.

b Data indicate the common name of the sequence.

c Data indicate the year in which the sequence was sampled.

d Data indicate the country/province in which the sequence was sampled. BW, Botswana; ZA/gp, South Africa/Guateng Province; ZA/kz, South Africa/KwaZulu-Natal Province; MW, Malawi; ZA/nw, South Africa/North West Province; TZ, Tanzania; ZM, Zambia.

e Data indicate whether the sequence represents the transmitted/founder (TF) that established homogeneous infection (F, false; NA, not available; T, true).

f Data indicate the infection stage at the time of sampling (E, early [A1 or A2]).

g Data indicate the presence of a potentially N-linked glycosylation sequon motif at the site(s) listed.

h Length data indicate the number of amino acids in the hypervariable region(s). aa, amino acids.

i Charge data indicate the sum of amino acid charges in the hypervariable region(s).

Table 2 summarizes the IC_50_ neutralization titers by 16 bnAbs. In the candidate panel, ZM233M and Ce703010010_C4 were resistant only to PGT128. Another Env, Ko243, was sensitive to all bnAbs shown. The selection of Envs sensitive to specific bnAb families is evident in the last three rows (Table 2).

**TABLE 2:** Clade C panel antibody neutralization IC_50_ titers^*a*^

Env accession no.^*b*^	Env name	Antibody neutralization IC_50_ titer (μg/ml) for indicated bnAb specificity															
		V2g					V3g				CD4bs					MPER	
		PGT145	PGDM-1400	PG9	CAP256-VRC26.08	CAP256-VRC26.25	PGT121	PGT128	10.1074V	10.1074	VRC07	VRC07-523	3BNC117	VRC01	VRC13	10E8	4E10
FJ443533	Ce703010010	**0.003**	**0.001**	**0.01**	**<0.0003**	**<0.0003**	**0.008**	>10	**0.008**	**0.031**	**0.025**	**0.01**	**0.041**	**0.11**	**0.25**	**0.772**	**5.2**
DQ388517	ZM233 M	**0.008**	**<0.001**	**0.002**	**<0.0003**	**<0.0003**	**2.809**	>10	**0.051**	**0.058**	**0.228**	**0.035**	**0.13**	**1.67**	**0.045**	**0.259**	**1.2**
DQ422948	ZM215F	>50	**0.008**	**0.02**	>25	>25	**0.01**	**0.06**	**0.006**	**0.028**	**0.058**	**0.041**	**0.018**	**0.17**	**0.365**	**0.067**	**0.4**
HM215307	3728	31.724	>50	**0.13**	**0.0004**	**<0.0003**	**3.783**	>10	>20	>20	**0.024**	**0.007**	**0.02**	**0.06**	**0.026**	**0.153**	**1.6**
KF114892	Ko243	**0.015**	**0.006**	**0.14**	**1.061**	**<0.0003**	**1.362**	**0.04**	**0.505**	**0.867**	**0.357**	**0.113**	**0.314**	**0.67**	**8.675**	**1.643**	18.62
FJ444124	Ce704810053	**3.398**	**0.003**	**0.16**	**<0.001**	**<0.0003**	>20	**0.01**	>20	>20	**1.41**	**0.069**	**1.582**	>10	**0.789**	**0.28**	**2.9**
HQ615959	2759058	**1.852**	**0.062**	**0.13**	**0.004**	**<0.0003**	**1.169**	>10	>20	>20	**0.537**	**0.155**	**1.141**	**2.64**	>25	**1.287**	**2.89**
JN681246	So431	35.309	>50	**0.34**	>25	>25	**0.021**	>10	**0.577**	>20	**0.354**	**0.012**	**0.198**	**0.56**	**0.072**	**0.161**	**2.5**
KC154028	CAP382	**1.272**	**0.021**	**0.07**	**<0.0003**	**<0.0003**	**9.911**	**6.42**	**0.8**	**5.885**	>25	**0.372**	>20	>10	>25	**1.511**	18.15
FJ444612	Ce2103	**2.533**	**0.012**	**2.6**	**0.002**	**<0.0003**	>20	>10	>20	>20	>25	>25	>20	>10	**0.221**	**2.506**	17.98
KF114882	B005582	>50	>50	>10	>25	>25	**6.839**	**0.03**	**0.027**	**0.034**	>25	**2.378**	>20	>10	**0.328**	**0.382**	23.64
HQ595766	2969249	>50	>50	>10	>25	>25	>20	>10	>20	>20	**0.93**	**0.222**	**0.64**	**1.37**	19.391	**0.921**	10.83

a Values below 10 μg/ml appear in bold text. See Fig. 5c for the corresponding heat map.

b A table containing these data for all 200 Envs is available in the supplemental Material.

Data Set S1 in the supplemental material lists the properties summarized in Table 1 and Table 2 for all 200 Envs.

### Comparison with earlier panels.

Earlier work published in 2006 described a panel of 12 clade C Envs from South Africa and Zambia, selected from among 18 viruses which were all acquired by heterosexual transmission and represented acute or early infections (18). Their median collection date was June 2001 (range, June 1998 through June 2005; there is always an inevitable lag between sample collection and publication). The median collection date among viruses in the current clade C panel was 2007 (range, October 2002 through 2010; the month of sample collection was not reported for these data). The average pairwise distance (APD) on trees from aligned *env* nucleotide sequences is 9.2% greater for this panel (0.250) than for the 2006 clade C panel (0.229), and both values are lower than that determined for the global multiclade panel (0.330), as expected. Phylogenetic distances are significantly greater for the current panel than for the 2006 panel (*n* = 66 in both; two-sided Wilcoxon test, *P* = 0.00018) and reflect the more challenging conditions of the current epidemic and test conditions for vaccine efficacy trials. These trees (not shown) were computed using PhyML version 3 (19, 20) with the GTR+Γ4+I substitution model and were rooted on HXB2, though the distances to HXB2 were excluded from panel APD calculations. Both panels were designed to represent acute and early infection following heterosexual transmission. Because increasing southern African clade C diversity is associated with reduced cross-reactive neutralization between sera and circulating HIV strains (9), a more divergent, more contemporary clade C panel better reflects the modern state of the epidemic. Such samples are difficult to obtain, and it takes years to acquire and evaluate them experimentally, so an even more recent sampling to assess vaccine trials that are under way is infeasible.

We also compared bnAb neutralization titers from viruses in each panel and summarized neutralization data for the 2006 clade C panel (Fig. 5a) and the global panel (Fig. 5b) from the CATNAP database (21). For the previously published panels, we extracted data available from CATNAP as of May 2017 (http://hiv.lanl.gov/catnap). Data from Envs with multiple published results are summarized as the geometric mean IC_50_ among unique values. That is, if an assay had been published three times with the same value and once with another value, only the two distinct neutralization values were averaged. This was done to avoid biased estimates resulting from the use of data from papers that reproduce results from earlier papers without repeating the experiment. One Env (ZM233M) was included in both clade C panels, identified in the figure by an asterisk. The candidate 2017 clade C panel (Fig. 5c) that we described above is no less sensitive to known bnAbs and is intended as an update to the 2006 clade C panel, for sensitive and informative plasma screening.

By design, several Envs in this new clade C panel shared patterns of reactivity to members of distinct bnAb classes. For example, B005582 is particularly sensitive to V3-glycan (V3g) bnAbs, Ce2103 to V1/V2-glycan (V2g) bnAbs, and 2969249 to CD4bs bnAbs (Fig. 5c). Detecting neutralization in plasma samples that have responses to one or more of these viruses would provide clues about antibody specificities therein and would provide information for follow-up experiments that map specificities or isolate monoclonal antibodies.

## DISCUSSION

To enhance scientific rigor, improve reproducibility, and unify efforts against HIV diversity, the use of standardized reference reagents for immunological assays is highly beneficial. Standardized reagents enable comparisons between different studies. We have described selection of standardized virus panels from HIV-1 clade C for several anticipated types of investigation, which include screening large numbers of sera from vaccinees for immune-induced neutralization responses and characterizing the magnitude and breadth of neutralization responses by newly isolated monoclonal antibodies.

Guided by the anticipated uses for these panels, we have described practical selection criteria, which utilize available information to obtain appropriately representative Env panels. We have described the use of hierarchical clustering and a simple but elegant downselection method to identify subsets of 100 and 50 clade C Envs from a panel of 200 well-characterized viruses. The panels performed better than randomly selected panels at characterizing magnitude-breadth distributions in aggregate across 16 bnAbs. For particular bnAbs, rather than the overall aggregate, moderate to almost no deviation appeared between the magnitude-breadth distributions revealed by our downselected panels and the full set of 200 Envs. This suggests that the smaller virus panels can be used in place of the full set to characterize bnAb magnitude-breadth distributions. Consequently, the use of smaller virus panels accelerates the rate at which bnAbs can be characterized. To avoid bias in favor of some bnAbs and against others, use of even smaller, 12-Env panels in magnitude-breadth studies is not recommended.

We used PCA of 16 bnAb IC_50_ neutralization titers to project 200 Env-pseudotyped viruses onto simplified coordinate systems for computationally guided Env selection. Using this representation, we identified a panel of 12 viruses that covered diverse bnAb sensitivity profiles on reduced dimensions. During panel selection, iterative refinement ensured that the 12 had a representative range of sensitivity to 30 chronic plasma samples.

We also tried automated methods (downselection, lasso, *k*-medoids) but favored the panel identified with computational guidance, because it does not merely reiterate the plasma neutralization continuum. The diversified detection strategy embodied by the candidate panel may therefore utilize limited sample materials more effectively than the automatically chosen Env sets, each of which contains closely related, and therefore redundant, neutralization profiles.

Clade-specific panels may be better able to detect relevant neutralization responses than nonspecific panels. In a previous study that tested South African plasma samples from individuals with C clade infections from the CAPRISA cohort, a panel of tier 2 clade C viruses showed greater sensitivity to neutralization than tier 2 virus panels from clades A and B (22). Similar findings have been reported in other studies (18, 23). We will not know how the two panels will compare with vaccinee sera until there is a vaccine that generates some measurable activity against tier 2 viruses. The earliest success at generating tier 2 virus neutralization could reflect partially matured bnAbs, and it is not known how these immature bnAbs might be differentially detected with clade-specific versus global virus panels.

On the other hand, we do not necessarily expect the candidate panel to perform “better” than the 2006 panel with HIV-1 sera. In fact, some of our previous data suggest the panels could perform similarly (12). The underlying scientific issue concerns potential differences in panel performance with vaccine-elicited antibodies, which cannot be assessed at the moment, because no vaccine yet tested elicits sufficient tier 2 virus neutralization responses. With this in mind, our goal was to design a panel of clade C viruses that are more contemporary and are selected on the basis of more-robust analysis methods to ensure the best possible representation of the current epidemic in southern Africa. The 2006 panel did not use neutralization phenotype data to guide its selection but instead included what was known and available at the time regarding Env genetic variation and reported neutralization assay results for the selected panel. We incorporated neutralization phenotypes throughout panel selection and selected from a very large, clade-specific neutralization panel. We expect the useful phenotypic characteristics of this new panel to emerge in subsequent work.

Our panel of 12 C clade Envs is intended as an update to the panel reported in 2006 (18). The 2006 panel was selected from a small subset through convenience sampling, whereas the 2017 panel was rationally selected from a much larger collection of viruses. The 2017 clade C panel contains more recently sampled Envs, deliberately includes sensitivity profiles that are characteristic of the currently known bnAb families, and includes greater genetic diversity than the earlier panel. This is important, because within-clade cross-reactive neutralization tends to decrease as genetic distance increases (23). Also, to help identify weak clade-specific responses without detecting the nonspecific antibody neutralization that is typical of a tier 1 response (24), the candidate panel includes a range of plasma sensitivities and favors neutralization-sensitive Envs without inclusion of known tier 1 Envs. Consequently, the 2017 clade C panel should be more informative and may be more sensitive than the 2006 clade C panel.

While we think that the candidate screening panel might provide hints about antibody specificities in plasma samples, it is intended for screening and not for epitope mapping, which would be performed to characterize samples that give positive test results for tier 2 neutralization activity. Further analysis would be needed to differentiate between possible specificities in a serum, and “next-generation” fingerprinting methods (25) could be useful for such purposes.

In contrast to the 12-virus global panel of multiclade viruses described in an earlier publication (12), we planned these panels to be used for screening sera and bnAbs from vaccinees where clade C infections predominate and clade C vaccines are being tested. We did not formulate a single quantitative metric to choose the virus panels proposed here for standardization. Instead, we considered a range of current needs for standardized reagents and selected sets of Envs that together satisfied these needs as we thought best. An extremely large number (6 × 10^18^) of alternative 12-Env panels is possible. We have described several methods to select useful sets of sequences that are intended to represent diversity in a large neutralization assay panel (6,000 plasma ID_50_ and 2,600 antibody IC_50_ titers). The Env panels we propose are reasonably representative of the diversity of the population from which they were chosen, by several different criteria. They represent distinctive bnAb sensitivity patterns and generally reflect the diversity of neutralization responses seen among sera from infected individuals.

HIV-1 clade C, which constitutes about half of all infections worldwide at present, represents formidable genetic diversity. As long as virus evolution continues, the ability to induce and detect immune responses against this highly diverse pathogen will be of sustained significance.

## MATERIALS AND METHODS

The CAVIMC-CAVD HIV-1 Clade C Virus Neutralization Phenotype Study was reviewed and approved by the research ethics committee of the Faculty of Health Sciences of the University of Cape Town (168/2007; 513/2012). All participants provided written informed consent for study participation (9).

Neutralization titers were determined with the TZM-bl luciferase assay previously described (26, 27) to test 200 recently described Envs against 16 bnAbs and plasma samples from 30 chronic infections (9). Antibodies studied included five CD4 binding-site (CD4bs) bnAbs, VRC01 (28, 29), VRC07 (30), VRC07-523 (31), VRC13 (32), and 3BNC117 (33); four V3-glycan (V3g) bnAbs, PGT121 (34), PGT128 (34), 10-1074 (35), and 10-1074V (35); five V1/V2-glycan (V2g) bnAbs, PGT145 (34), CAP256-VRC26.08 (36), CAP256-VRC26.25 (37), PG9 (38), and PGDM1400 (39); and two membrane-proximal external region (MPER) bnAbs, 10E8 (40) and 4E10 (41). We sometimes refer to CAP256-VRC26.08 and CAP256-VRC26.25 as VRC26.08 and VRC26.25 for brevity here.

### Magnitude-breadth panels (50 and 100 Envs).

Large virus panels are useful to characterize the magnitude and breadth of neutralizing antibodies, but panel size limits the rate at which results can be obtained. Using large neutralization panels is very expensive and may consume excessive reagent resources. The trade-off is that excessively small panels may not contain a sufficient amount of the information needed to make fair assessments across different bnAbs. We therefore downselected representative sets of 50 and 100 Envs to facilitate studies of antibody magnitude and breadth.

We used a simple strategy to select subsets of viruses that represent the diversity of responses in the full set. To compare Env profiles, we used the Euclidean distance between vectors of 16 bnAb 50% inhibitory concentration (IC_50_) neutralization titers and then hierarchically clustered the 200 Envs. We weighted the resulting dendrograms by geometric mean IC_50_ to obtain a gradient from most sensitive Env to least sensitive Env (within the constraints of the dendrogram branching structure). We used Ward's method (15) for hierarchical clustering but also considered other methods. A simple downselection procedure alternated through the rows of the dendrogram-ordered neutralization heat maps by inclusion of one Env and exclusion of the next. We repeated this procedure to downselect from the full panel of 200 Envs and obtain smaller panels comprised of 100 or 50 Envs. We kept the same row and column order in neutralization panels during downselection rather than recluster and reorder.

For each of 16 bnAbs, we compared the magnitude-breadth distributions of the full panel of 200 clade C Envs with those of the downselected panels. The area between curves (ABC) quantified the difference between the two cumulative distribution functions. We used resampling to evaluate further the ABC values from downselected panels. Random panel selection characterized the null distribution of ABC values to reveal whether dendrogram-based downselection gave significantly lower values than could be obtained by chance. We randomly sampled 100-Env panels from all 200 Envs (without replacement) 10^4^ times. From each of these, we also sampled a random 50-Env panel. We computed resampled ABCs against the distribution from 200 Envs and compared these with values from the downselected panels.

We repeated the downselection procedure to obtain an even smaller panel of 12 Envs.

### Serum screening panel (12 Envs).

For the purpose of screening sera from vaccinees, we tried several approaches to select a small panel of viruses, intended to include Envs sensitive to a variety of neutralizing antibodies and sera. This smaller “candidate” panel includes 12 pseudoviruses chosen to detect neutralization responses in vaccinees and to suggest possible antibody specificities therein. Virus selection was guided by neutralization titers from assays against bnAbs and chronic sera from each of 200 Envs. Tier phenotyping (24) of these Envs demonstrated 1.3% tier 1A (*n* = 2), 8.5% tier 1B (*n* = 17), 75% tier 2 (*n* = 150), and 15.5% tier 3 (*n* = 31) Envs. We excluded the two tier 1A Envs and three highly sensitive tier 1B Envs (geometric mean 50% infective dose [ID_50_] titers above 250 reciprocal dilutions) from panel selection because they seemed unlikely to be useful in distinguishing the protective responses from those that are nonprotective (13, 14).

Our strategy was to select Envs using bnAb IC_50_s to ensure that all specificities were included and to compare the data to ID_50_s from plasma samples from patients with chronic infection. We used principal-component analysis (PCA) to simplify high-dimensional data from neutralization assays by projecting them onto fewer dimensions. The overall effect of dimension reduction is achieved by decomposing correlations among the data into principal components (42). This approach has recently been used for unsupervised learning to characterize high-dimensional immunological data from HIV Env antigens (43).

In a computationally guided procedure, we iteratively selected candidate Env panels and then reviewed their distributions in lower-dimensional projections of bnAb IC_50_s. Where the candidate panel contained clusters of Envs rather than dispersed Envs, different Envs were chosen to increase the separation between them and to increase coverage of known specificity profiles with the least overlap possible. This approach enabled us to select 12 candidate Envs that captured the diversity of known bnAb specificities, while ensuring low redundancy among the specificity profiles. We think it is important to sample the diversity of natural antibody responses to heterologous virus isolates because we do not know *a priori* the nature of the neutralizing antibodies that may be elicited and that may correlate with vaccine-mediated protection. We compared this PCA-guided strategy to automatic selection using lasso (12, 44, 45) and a *k*-medoids clustering strategy (via the pam package in R, version 2.0.5), in addition to the downselection procedure developed for larger panels.

All analysis was done using R (versions 3.3.0 through 3.4.0). We computed Env hypervariable loop lengths and net charges as described previously (9, 23).

## Supplementary Material

Supplemental material
