# Supplementary material for: Panels of HIV-1 Subtype C Env Reference Strains for Standardized Neutralization Assessments
Source: J Virol. 2017 Sep 12;91(19):e00991-17. doi: 10.1128/JVI.00991-17 (PMC5599761; doi:10.1128/JVI.00991-17)
Supplement: Supplemental material [file supp_91_19_e00991-17__index.html]

Supplemental material 

# Panels of HIV-1 Subtype C Env Reference Strains for Standardized Neutralization Assessments

## Supplemental material

- Supplemental file 1 -

  Fig. S1 (Heatmaps of bnAb IC50s to illustrate down-selection from hierarchically clustered row ordering to obtain 12 Envs.)

  Fig. S2 (Magnitude-breadth distributions from 12-Env panels.)

  Fig. S3 (Principal component analysis of bnAb IC50s.)

  Fig. S4 (Comparison of neutralization ID50 clustering methods among dendrograms from 200 Envs and 30 chronic plasmas.)

  Fig. S5 (Plasma neutralization ID50s for 195 Envs against 30 chronic plasmas.)

  Fig. S6 (Comparison of plasma neutralization ID50s for four 12-Env panels.)

  Table S1 (Comparison of area between curves from the cumulative magnitude-breadth distributions of all 200 Envs with either down-selected or randomly selected panels.)

  Data Set S1 (Properties of the 200 clade C Envs studied.)

  PDF, 505K
- Supplemental file 2 -

  Data Set S1 (Properties of the 200 clade C Envs studied.)

  XLSX, 78K
