# Supplementary material for: Panels of HIV-1 Subtype C Env Reference Strains for Standardized Neutralization Assessments
Source: J Virol. 2017 Sep 12;91(19):e00991-17. doi: 10.1128/JVI.00991-17 (PMC5599761; doi:10.1128/JVI.00991-17)
Supplement: Supplemental material [file JVI.00991-17_zjv999182949s1.pdf]

Panels of HIV-1 Subtype C Env Reference Strains for Standardized Neutralization Assessments

Peter Hraber, Cecilia Rademeyer, Carolyn Williamson, Michael S. Seaman, Raphael Gottardo, Haili Tang, Kelli Greene, Hongmei Gao, Celia LaBranche, John R. Mascola, Lynn Morris, David C. Montefiori, Bette T. Korber

SUPPLEMENTAL MATERIALS

Magnitude-Breadth Panels (50 and 100 Envs)

To select sets of 100 and 50 Envs, we used down-selection based on relative Env ordering in a weighted dendrogram (**Figure 2**). Area between curves (ABC) measures the difference between two cumulative distribution functions. Decreasing the panel size from 100 to 50 Envs roughly doubled the ABC (**Table S1**). This affects some bnAbs more than others. In the worst case, VRC13 shows more Envs neutralized (IC50 below 25 μg/ml) for 50 Envs (80%) than 100 (75%) or 200 Envs (71%). Conversely for VRC01, the proportion of IC50s below 10 μg/ml is 22% of 200, 25% of 100, and 30% of 50 Envs.

A simple test of randomly sampling 100- and 50-Env panels suggests that some bnAbs are easier to characterize than others (**Table S1**). In the worst cases, VRC13 and 10E8 were better represented by having lower ABC values among 67% and 85% of 10^4 randomly chosen panels than the dendrogram-selected panels. In contrast, aggregate performance of the selected panels, taken as the total, mean, or median ABC across all 16 bnAbs, was much better than random, with 5.7%

and less of the random panels having lower ABCs than selected panels (**Table S1**). This simple comparison with the null distribution suggests that dendrogram-based panel down-selection is not merely a random sampling process.

Serum Screening Panel (12 Envs)

In addition to computationally guided panel selection using principal components analysis (PCA), we tried several automated methods to obtain 12-Env panels: down-selection, lasso, and *k*-medoids. We used the down-selection approach to obtain a set of 12 Envs that span the range of neutralization sensitivities (**Figure S1**). In this approach, one of the down-selected Envs had been excluded from panel consideration because of Tier 1 neutralization susceptibility, and was therefore also excluded from down-selection.

The bnAb magnitude-breadth distributions with both the 2006 and down-selected 12-Env panels show large deviation (area between curves, ABC) from the full set of 200 Envs (**Figure S2**). ABC increased disproportionately with smaller panels. The average ABC per bnAb was 3.1 times greater for 12 down-selected Envs than 50 and 4.7 times greater for the 2006 panel than 50 Envs. Whereas down-selection from 100 to 50 Envs increased total ABC by a factor of 2.1, down-selection from 50 to 12 increases total ABC by a factor of 2.8. The 2006 12-Env panel increased ABC by 3.8 times, relative to 50 Envs.

The 12-Env panel we selected with computational guidance (“Candidate Panel” in **Figure S2**) did not perform better to characterize bnAb magnitude-breadth distributions. Again, large devia-

tions appear from the distribution with all 200 Envs (**Figure S2**). The average ABC per bnAb was 3.6 times greater for the candidate panel than for 50 Envs. Down-selection from 50 to the 12-Env panel increased total ABC by a factor of 3.9.

Further, a bias in favor of some bnAbs and against others becomes clear with 12 rather than with 50 or 100 Envs. (ABC variation increases.) For example, the V2 glycan bnAbs PGDM1400 and VRC26.25 have inflated magnitude-breadth profiles relative to the V3 glycan bnAbs PGT128 and 10.1074, though this bias would be reduced or eliminated with the larger Env panels we describe in **Figure 3**. Together, these findings suggest that 12-Env panels are less suitable for bnAb magnitude-breadth characterization than larger panels of 50 or 100 Envs.

In addition to down-selection (described above) and two automated methods (*k*-medoids and lasso, detailed below), we used a computational approach to guide the selection of the "candidate" 12-Env panel, based on neutralization assay results. Iterative refinement of the candidate panel avoided Envs with extremely high and low geometric mean ID50s, and instead favored a range of intermediate sensitivity to plasma neutralization. We also ensured that each plasma could detectably neutralize at least two panel Envs. We iterated through four rounds of candidates, to ensure we had covered the diversity of plasma and bnAb responses. IC50 titers were inverted as 1000/IC50 and $\log_{10}$-transformed before analysis so more potent outcomes weighed more heavily than interactions without neutralization. Before analysis, the input data were centered by subtracting the mean and standardized by dividing against the variance, i.e. the correlation matrix was used as input. The R function prcomp did the analysis.

We further evaluated alternative sets of 12 Envs, one obtained by partitioning about medoids (1). Rather than use a greedy initial seeding, which quickly generates an approximate but suboptimal solution (2-4), we seeded the partitioning to begin by building clusters around the 12 panel Envs, then let the pam algorithm proceed iteratively. To enable combining serological and monoclonal antibody data, we constructed a data matrix that combined serum ID50s and bnAb IC50s by rank transforming values. That is, neutralization titers against each serum were ranked from lowest (<20 reciprocal dilutions, i.e. detectable neutralization) to highest (most potent) ID50. Conversely, we ranked bnAb IC50s from highest (>10 µg/ml, i.e. weakest potency) to lowest (<0.001 µg/ml). For tied values, we assigned ranks to reflect the number of unique neutralization titers for each antibody or plasma sample. For example, serum ID50 values of 10, 10, 10, 50, 100, 100 would be transformed to 1, 1, 1, 2, 3, 3. Similarly, antibody IC50 values 10, 10, 10, 5, 0.001, 0.001 would also be transformed to 1, 1, 1, 2, 3, 3. This approach to transforming serum and antibody neutralization values is also used for neutralization fingerprinting (5). Clustering used Euclidean distances between vectors of ranked neutralization scores. Envs to represent 12 medoids were computed by pam (version 2.0.5) in R, initially set to the candidate 12-Env panel.

We also used the lasso (least absolute shrinkage and selection operator) penalized regression method (6) to select a panel of Envs to model the geometric mean plasma ID50 neutralization titers, in a manner described previously (7). Unlike the approach used previously, we did not standardize variables to unit variance because they were all measured in terms of the same units, i.e. reciprocal dilutions. Neither did we utilize the area under the curve (AUC) from the dilution series as before (7), but rather the ID50 titers. Lasso is a penalized regression procedure for feature selection, which requires that the sum of the absolute values of regression coefficients be

less than some given constant value (6, 8). This constraint forces most regression coefficients to be zero-valued, and the non-zero coefficients are taken to indicate inclusion. Systematically varying stringency of the constraint gives a succession of candidate solutions, each of which accounts for a greater proportion of deviance (6, 8).

PCA projects the data onto simplified coordinate systems, whose axes have useful properties. The axes are orthogonal (i.e. perpendicular to each other), comprised of bnAb families (i.e. combinations of related bnAbs are weighted for scoring Envs on each axis), and ranked to account for as much data variation as possible (i.e. the first axis explains more variance than the second, etc.).

Figure S3 summarizes PCA of bnAb IC50s. Candidate panel Envs (the 12 chosen with computational guidance) are identified with black asterisks behind them. The dispersion of selected panel Envs, i.e. lack of overlap, in this low-dimensional representation was used to choose the candidate panel. For comparison, Env panels identified by *k* medoids (pam) are marked by blue asterisks, and the lasso-selected Envs by red asterisks (**Figure S3**). The first two principal components together explain about half the variance (47.6%) in the bnAb IC50 data. These two axes were strongly associated with Env sensitivity to bnAbs. With Env points colored to show the number of bnAbs with neutralization titers below 10 μg/ml as in **Figure 4a**, a tendency for lower sensitivity is clearly apparent in the transition from the lower-right to the upper-left quadrant (**Figure S3a**).

To help interpret the projection defined by principal components axes, **Figure S3b** shows the contribution of each bnAb, in the manner of a biplot (9, 10). The lower-right quadrant of PC1 and PC2 is predominantly associated with the five CD4bs bnAbs VRC01, VRC07, VRC07.523, VRC13, and 3BNC117 (**Figure S3b**). The second principal component reflects differences in response to V3g Abs PGT121, PGT128, 10-1074, and 10-1074V (**Figure S3b**, lower-left quadrant) versus the V2g Abs PG9, VRC26.08, and VRC26.25, plus PGT145 and PGDM1400 (**Figure S3b**, top-right).

Including the third principal component added 17% of the variance, for a cumulative total of 63.6%. That is, three dimensions (orthogonal axes) explain nearly two-thirds of the variance in the matrix of 3200 IC50 neutralization titers. Projecting the viruses onto this and the first axis (**Figure S3c**) shows a gradient similar to that seen above on PC Axes 1 and 2, though the bolus of Envs in the lower-right quadrant of **Figure S3a** associated with high CD4bs susceptibility is less prominent. Rotating the loadings of bnAbs onto orthogonal axes suggests that the four bnAb categories (CD4bs, V3g, V2g, and MPER) are distributed in the reduced-dimensional space as vertices of a tetrahedron, or four-faced pyramid (**Figure S3, b** and **d**).

Including the fourth principal component adds 9% of the total variance and separates the MPER antibodies 10E8 and 4E10 into a cluster of their own (not shown). The number of bnAbs with IC50 below 10 μg/ml was not distinctly associated with PC Axis 4. Using more than four axes yielded diminishing returns of variance explained by each (e.g. 5.1% for a fifth axis) and did not suggest clear interpretation, aside from stratifying antibodies with shared specificities into smaller groups (not shown).

Envs from the candidate, lasso, and *k*-medoids panels are distributed over the reduced-dimension bnAb neutralization space. Any set might possibly be used to suggest antibody specificities in plasma. One clear difference between the candidate panel and the two automatically selected panels is that the latter two both include a single Env that is sensitive to nearly every bnAb. They appear in the lower-right quadrant of **Figure S3a**. We did not pick Envs from this region during computationally guided selection of the candidate panel, because it seemed they might not contain specific neutralization information, being sensitive to multiple bnAb types.

Reviewing the projection of Envs onto principal components suggests the candidate Env panel should be at least as informative as the *k*-medoids set at suggesting bnAb specificities in sera (**Figure S3**). This is because the selected Envs (red asterisks) are distributed over the principal component axes and all 12 Envs were neutralized by at least nine bnAbs. In contrast, one of the *k*-medoids Envs (blue asterisks) was neutralized by fewer than nine bnAbs. Another *k*-medoid Env was neutralized by all 16 bnAbs, and is located in the lower-right quadrant given by the first two principal components (**Figure S3a**), where it would be unlikely to suggest any specific bnAb activity, because of its high overall susceptibility.

Regarding the choice of methods for the hierarchical clustering used in **Figure 6**, two methods (median and centroid) yielded negative branch lengths and were disregarded. Each of the other six gave clustering solutions that grouped related virus and serum neutralization profiles in slightly different ways. To compare results obtained from different hierarchical clustering methods, we used the dendextend package and computed pairwise correlation coefficients from co-

phenetic distances, which summarize the degree of dissimilarity at which two observations are combined into one cluster (11). Such coefficients range from zero (no correlation) to one (perfect association). **Figure S4** summarizes the relatedness of plasma ID50 clusters from each method, for each possible pair. The correlations between virus clusterings (**Figure S4a**) are generally lower than for serum clusterings (**Figure S4b**), because the former contains 200 leaves and the latter only 30. The large variation in coefficients among clustering methods indicates that the different methods can yield very different results. Unless there is a reason to choose one over the others beforehand, it may be worth trying alternative methods, rather than choose only one. When we did this and reviewed the results (not shown), we thought Ward's method with squared Euclidean distances served well to group related neutralization profiles together. This grouping was useful to identify shared responses that may not otherwise be apparent, such as from sorting Envs and sera by their geometric mean ID50 titers.

A heatmap summarizes neutralization ID50s (**Figure S5**) from plasma donors with chronic viremia against clade C Envs. The variation of plasma potencies is evident. Plasmas are sorted from highest (left column) to lowest (right) geometric mean ID50. Env responses range from most neutralization sensitive (top row) to least (bottom). The twelve selected viruses are distributed throughout this interval, rather than being clumped or concentrated, and reflect the distribution of virus neutralization sensitivities shown in **Figure 4c**. In contrast, the *k*-medoid Envs (in blue) are more dispersed than the selected candidate panel Envs (in black), and reflect even greater variation of neutralization sensitivities to plasma. The lasso-selected set (in red) includes a progression from sensitive through moderately resistant Envs, but excludes nearly all of the lower half of the distribution of neutralization sensitivities (**Figure S5**). In both the candidate and *k*-

medoid sets, each of the twelve selected Envs was neutralized by at least two of the 30 plasmas sampled. In contrast, among lasso-selected Envs, only one Env was neutralized in the case of two plasma samples, which could suggest a greater risk for false negatives (**Figure S5**). For these reasons, we favor the computationally guided candidate panel over panels selected automatically by *k*-medoids or the lasso.

The down-selected 12-Env panel was able to detect serum neutralization responses less often than the candidate 12-Env panel (**Figure S6**). For the down-selected panel, 67.2% of 360 serum assays showed quantifiable ID50 neutralization titers, versus 72.5% of 360 assays (same 30 sera, 12 different Envs). The down-selected panel therefore seems less able to detect Tier 2 responses than the candidate panel.

The lasso and *k*-medoids panels reflect the continuum of variation in plasma sensitivities seen for the full set of 200 Envs (**Figure S6)**. In contrast, plasma neutralization responses for the candidate panel are more roughly distributed (**Figure S6**). This suggests the candidate panel does not merely reflect the average neutralization sensitivity of the full 200-Env set. Computationally guided selection avoided individual Envs that were sensitive to all bnAb specificities. The automatically selected panels represent overall plasma behavior. Consequently, they contain more redundancy and sacrifice the opportunity to suggest what neutralization specificities may be present in plasma samples, which are limited in volume and may be very difficult to obtain.

SUPPLEMENTAL FIGURE LEGENDS

Figure S1. Heatmaps of bnAb IC50s to illustrate down-selection from hierarchically clustered row ordering to obtain 12 Envs. (**a**) All 200 Envs, as depicted in **Figure 2a**. (**b**) Down-selected Envs. Arrows identify corresponding rows in the full data set.

Figure S2. Magnitude-breadth distributions from 12-Env panels. This representation follows **Figure 3** but illustrates three 12-Env panels, the down-selected set (magenta), the 2006 clade C panel (12) illustrated in **Figure 5a** (red), with CATNAP data as described in the main text, and the candidate panel developed using computational guidance (cyan).

Figure S3. Principal component analysis of bnAb IC50s. Five Envs with geometric mean ID50s above 250 were excluded. (**a**) The 195 clade C Envs projected onto first two principal components (PC). Colors follow **Figure 4a** to show the number of bnAb IC50s below 10 μg/ml. The candidate panel of 12 Envs selected with computational guidance (black), *k*-medoid Envs (blue), and lasso-selected Envs (red) are indicated with asterisks. The proportion of variance among the input data associated with PC 1 was 23.9% and 23.7% for PC 2. (**b**) Arrows indicate the rotations (loadings, i.e. eigenvectors) for each bnAb on first two principal components. Colors indicate shared bnAb specificities. (**c**) Envs projected onto PC 1 and 3, which flips the above plot along the *x*-axis by 90 degrees. (**d**) bnAb loadings on PC 1 and PC 3. Together, the first three principal components (shown) include 63.6% of the variance among bnAb IC50s. The fourth and fifth axes (not shown) add 9% and 5.1% (respectively) to the total variance explained, which suggests diminishing returns from adding axes.

Figure S4. Comparison of neutralization ID50 clustering methods among dendrograms from (**a**) 200 Envs and (**b**) 30 chronic plasmas. Clustering methods are named along the diagonal. Of the eight methods provided as options to the R hclust procedure, we summarize here the six that did not yield negative branch lengths. Cophenetic correlations were computed from cophenetic distances using Kendall's τ. A visual depiction appears above the diagonal, with circle diameter and color proportional to the correlation statistic in the corresponding cell below the diagonal. For example, the large circle in the top row corresponds to the correlation between cophenetic distances between clusters obtained by Ward's method on Euclidean (D) and Ward's method on squared Euclidean distances (D2).

Figure S5. Plasma neutralization ID50s for 195 Envs against 30 chronic plasmas. (Five Envs with geometric mean ID50s above 250 were excluded.) Rows were sorted from most to least sensitive virus, and columns were sorted from most to least potent plasma. Accession numbers per Env appear in the margins. The 12 candidate Envs selected with computational guidance are indicated by black text, down-selected with magenta, lasso with red, and the *k*-medoids set with blue text. The remaining 147 Envs are indicated by grey text. The histogram summarizes ID50 values as heatmap colors.

Figure S6. Comparison of plasma neutralization ID50s for four 12-Env panels. Down-selected Envs are depicted with magenta text, the lasso panel with red text, *k*-medoids panel with blue text, and finally the candidate panel, obtained by computationally guided selection.

REFERENCES

1. Kaufman L, Rousseew PJ. 2005. Chapter 2: Partitioning around medoids Finding groups in data: an introduction to cluster analysis. John Wiley and Sons, Hoboken.
2. Bang-Jensen J, Gutin G, Yeo A. 2004. When the greedy algorithm fails. Discrete Optimization 1:121–7.
3. Bendall G, Margot F. 2006. Greedy-type resistance of combinatorial problems. Discrete Optimization 3:288–98.
4. Cormen TH, Leiserson CE, Rivest RL, Stein C. 2009. Chapter 16: Greedy algorithms. Introduction to agorithms, third edition. MIT Press, Cambridge, MA.
5. Georgiev IS, Doria-Rose NA, Zhou T, Kwon YD, Staupe RP, Moquin S, Chuang GY, Louder MK, Schmidt SD, Altae-Tran HR, Bailer RT, McKee K, Nason M, O'Dell S, Ofek G, Pancera M, Srivatsan S, Shapiro L, Connors M, Migueles SA, Morris L, Nishimura Y, Martin MA, Mascola JR, Kwong PD. 2013. Delineating antibody recognition in polyclonal sera from patterns of HIV-1 isolate neutralization. Science 340:751–6.
6. Tibshirani R. 1996. Regression shrinkage and selection via the lasso. J R Stat Soc Series B Stat Methodol 1:267–88.
7. deCamp A, Hraber P, Bailer RT, Seaman MS, Ochsenbauer C, Kappes J, Gottardo R, Edlefsen P, Self S, Tang H, Greene K, Gao H, Daniell X, Sarzotti-Kelsoe M, Gorny MK, Zolla-Pazner S, LaBranche CC, Mascola JR, Korber BT, Montefiori DC. 2014. Global panel of HIV-1 Env reference strains for standardized assessments of vaccine-elicited neutralizing antibodies. J Virol 88:2489–507.
8. Friedman J, Hastie T, Tibshirani R. 2008. Regularization paths for generalized linear models via coordinate descent. J Stat Softw 33.

9. Gabriel KR. 1971. The biplot graphical display of matrices with application to principal component analysis. Biometrika 58:453–467.

10. Gower J, Lubbe S, le Roux N. 2011. Chapter 3: Principal component analysis biplots. Understanding biplots. John Wiley and Sons, West Sussex, UK.

11. Sneath PHA, Sokal RR. 1973. Numerical taxonomy: the principles and practice of numerical classification. Freeman, San Francisco, CA.

12. Li M, Salazar-Gonzalez JF, Derdeyn CA, Morris L, Williamson C, Robinson JE, Decker JM, Li Y, Salazar MG, Polonis VR, Mlisana K, Karim SA, Hong K, Greene KM, Bilska M, Zhou J, Allen S, Chomba E, Mulenga J, Vwalika C, Gao F, Zhang M, Korber BT, Hunter E, Hahn BH, Montefiori DC. 2006. Genetic and neutralization properties of subtype C human immunodeficiency virus type 1 molecular env clones from acute and early heterosexually acquired infections in Southern Africa. J Virol 80:11776–90.

13. Hraber P, Korber BT, Lapedes AS, Bailer RT, Seaman MS, Gao H, Greene KM, McCutchan F, Williamson C, Kim JH, Tovanabutra S, Hahn BH, Swanstrom R, Thomson MM, Gao F, Harris L, Giorgi E, Hengartner N, Bhattacharya T, Mascola JR, Montefiori DC. 2014. Impact of clade, geography, and age of the epidemic on HIV-1 neutralization by antibodies. J Virol 88:12623–43.

14. Rademeyer C, Korber B, Seaman MS, Giorgi EE, Thebus R, Robles A, Sheward DJ, Wagh K, Garrity J, Carey BR, Gao H, Greene KM, Tang H, Bandawe GP, Marais JC, Diphoko TE, Hraber P, Tumba N, Moore PL, Gray GE, Kublin J, McElrath MJ, Vermeulen M, Middelkoop K, Bekker LG, Hoelscher M, Maboko L, Makhema J, Robb ML, Abdool Karim S, Abdool Karim Q, Kim JH, Hahn BH, Gao F, Swanstrom R, Morris L, Montefiori DC, Williamson C. 2016. Features of recently transmitted HIV-1 clade C viruses that impact

antibody recognition: implications for active and passive immunization. PLoS Pathog 12:e1005742.

Table S1. Comparison of area between curves (ABC) from the cumulative magnitude-breadth distributions of all 200 Envs with either down-selected or randomly selected panels.

**bnAb**	**ABC 100**[1]	**Random 100-Env Panels with ABC ≤ ABC 100, %**[2]	**ABC 50**[3]	**Random 50-Env Panels with ABC ≤ ABC 50, %**[4]
PGT145	0.0497	5.91	0.1677	48.68
PGDM1400	0.05102	6.49	0.05956	0.31
PG9	0.04874	16.59	0.11719	39.90
VRC26.08	0.07443	22.56	0.12431	19.81
VRC26.25	0.0474	8.65	0.08719	10.22
PGT121	0.05145	11.14	0.09472	14.72
PGT128	0.03884	10.09	0.09306	25.56
10-1074	0.05356	15.74	0.0702	3.57
10-1074V	0.05462	8.94	0.10584	13.40
VRC07	0.04909	23.38	0.11881	54.02
VRC07.523	0.0336	3.19	0.06661	6.47
3BNC117	0.0353	3.03	0.14133	60.21
VRC01	0.04585	40.78	0.0701	28.73
VRC13	0.09035	67.34	0.2119	85.81
10E8	0.07448	82.79	0.1102	67.51
4E10	0.02196	1.05	0.08412	58.22
Sum	0.82041	0.02	1.72283	2.15
Mean	0.05128	0.02	0.10768	2.15
Median	0.0494	0.39	0.10028	5.68

1 Area between curves (ABC) quantifies the difference between curves from 100 and 200 Envs.
2 Of 10^4 random 100-Env panels, percentage with ABCs not greater than selected 100-Env panel.
3 Area between curves for 50-Env panel and all 200 Envs.
4 Percentage of 10^4 random 50-Env panels with ABCs not greater than the selected 50-Env panel.

Dataset S1. Properties of the 200 clade C Envs studied. GenBank accession numbers are listed with virus names and metadata (geographic source, year sampled, is transmitted-founder virus, and infection stage sampled, whether Early, Acute1, or Acute2) following Rademeyer et al. (2016), then an indication of which Envs are in the selected panels (in.12, in.100, in.50), and IC50 neutralization titers. Inequality symbols indicate censored IC50s. In addition to the 16 bnAbs reported in the main text, IC50 titers are listed for 2F5, 2G12, IgG1b12, and soluble CD4 (sCD4). Additional columns indicate the presence of potential N-linked glycosylation sies (PNGs) at HXB2-numbered positions associated with neutralization sensitivity or resistance (N332/N334, N293/N295, N130, N156/N160), then the length, number of PNG sites, and net charge of hypervariable loop regions V1, V2, V1/V2, V3, V4, and V5 (13, 14).

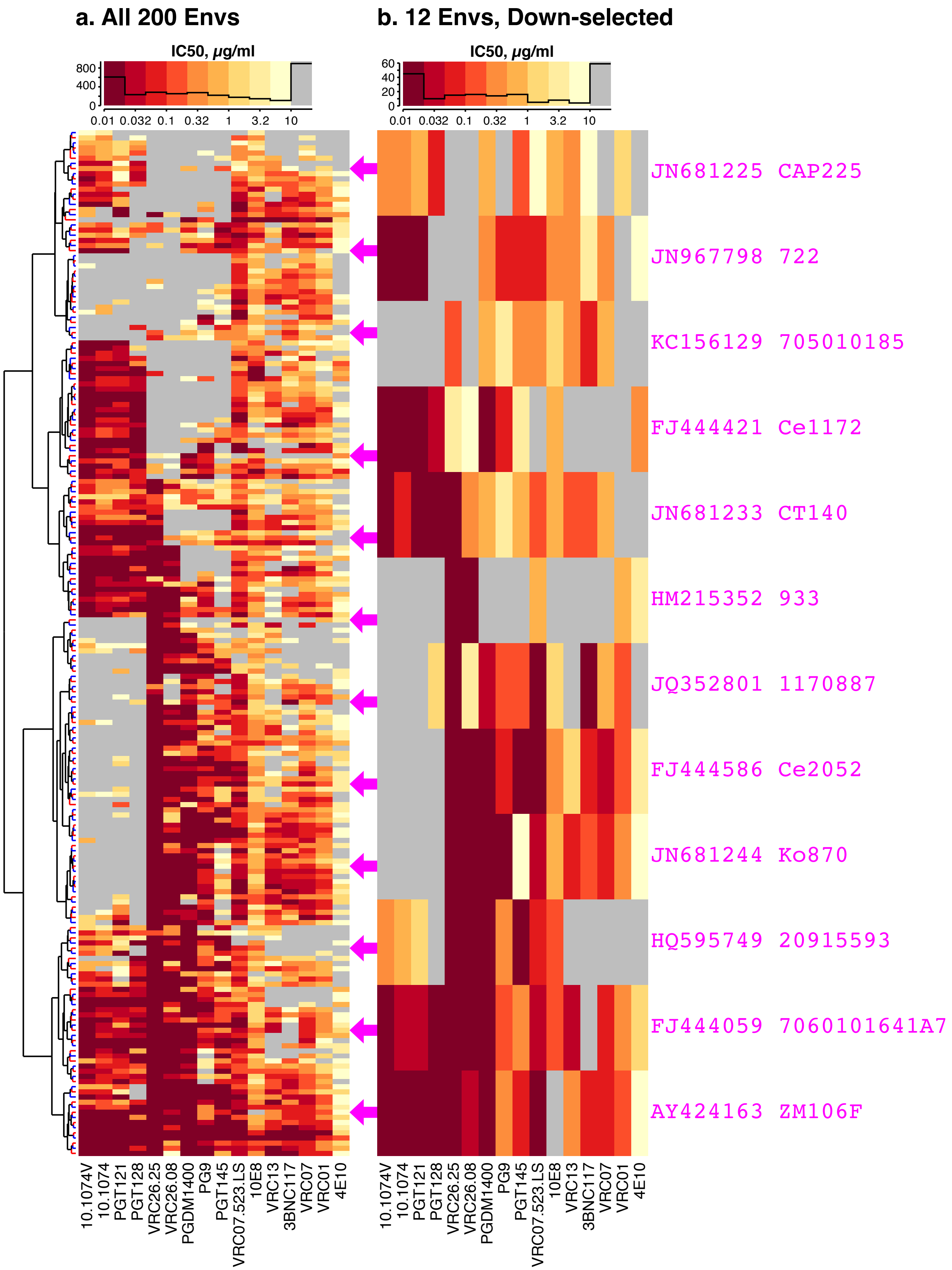
a. All 200 Envs
b. 12 Envs, Down-selected
IC50, μg/ml
800
400
0
0.01 0.032 0.1 0.32 1 3.2 10
IC50, μg/ml
60
40
20
0
0.01 0.032 0.1 0.32 1 3.2 10
JN681225 CAP225
JN967798 722
KC156129 705010185
FJ444421 Ce1172
JN681233 CT140
HM215352 933
JQ352801 1170887
FJ444586 Ce2052
JN681244 Ko870
HQ595749 20915593
FJ444059 7060101641A7
AY424163 ZM106F
10.1074V
10.1074
PGT121
PGT128
VRC26.25
VRC26.08
PGDM1400
PG9
PGT145
VRC07.523.LS
10E8
VRC13
3BNC117
VRC07
VRC01
4E10

Figure S1

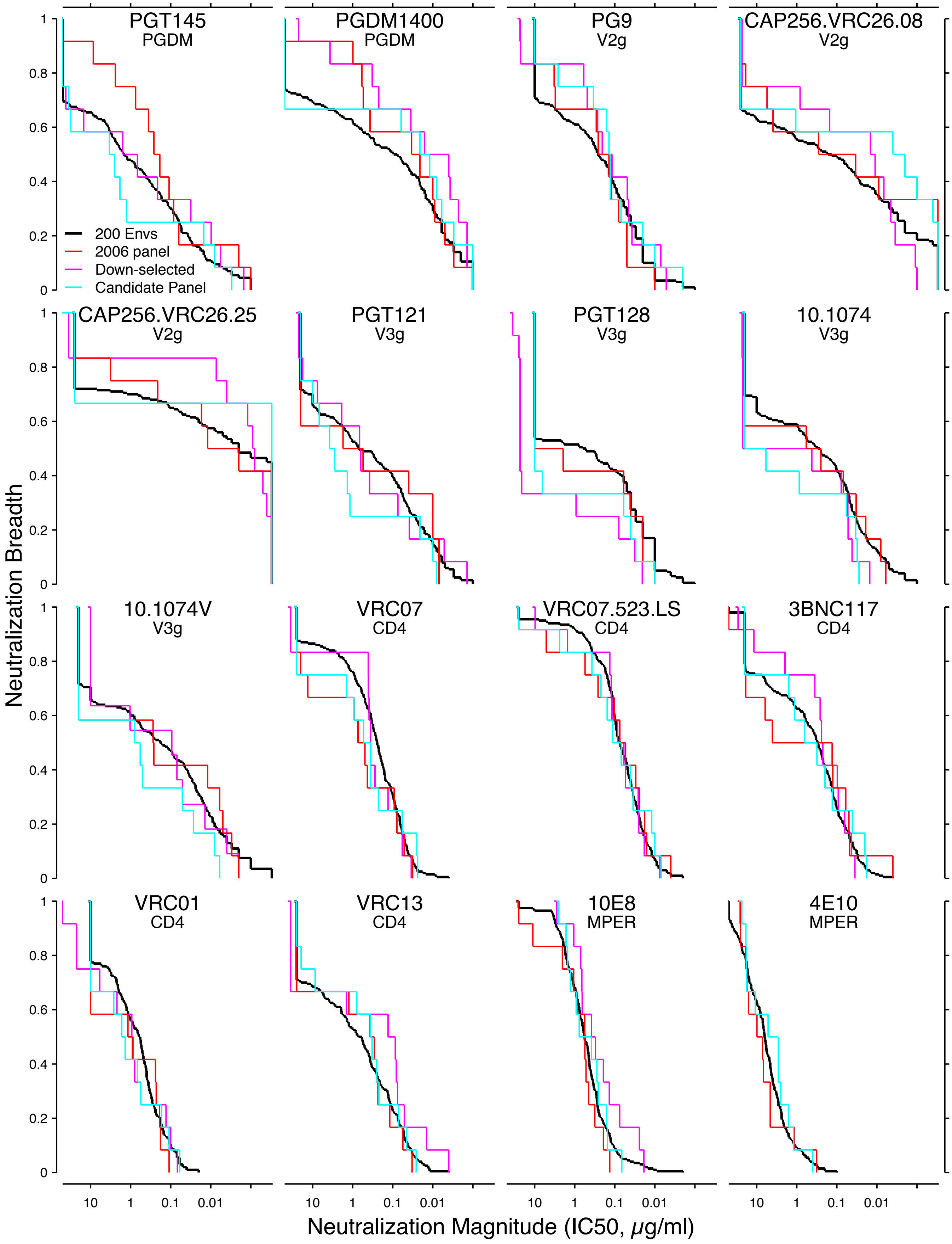

PGT145
PGDM
PGDM1400
PGDM
PG9
V2g
CAP256.VRC26.08
V2g
CAP256.VRC26.25
V2g
PGT121
V3g
PGT128
V3g
10.1074
V3g
10.1074V
V3g
VRC07
CD4
VRC07.523.LS
CD4
3BNC117
CD4
VRC01
CD4
VRC13
CD4
10E8
MPER
4E10
MPER
200 Envs
2006 panel
Down-selected
Candidate Panel
Neutralization Breadth
Neutralization Magnitude (IC50, μg/ml)
0
0.2
0.4
0.6
0.8
1
10
1
0.1
0.01

Figure S2

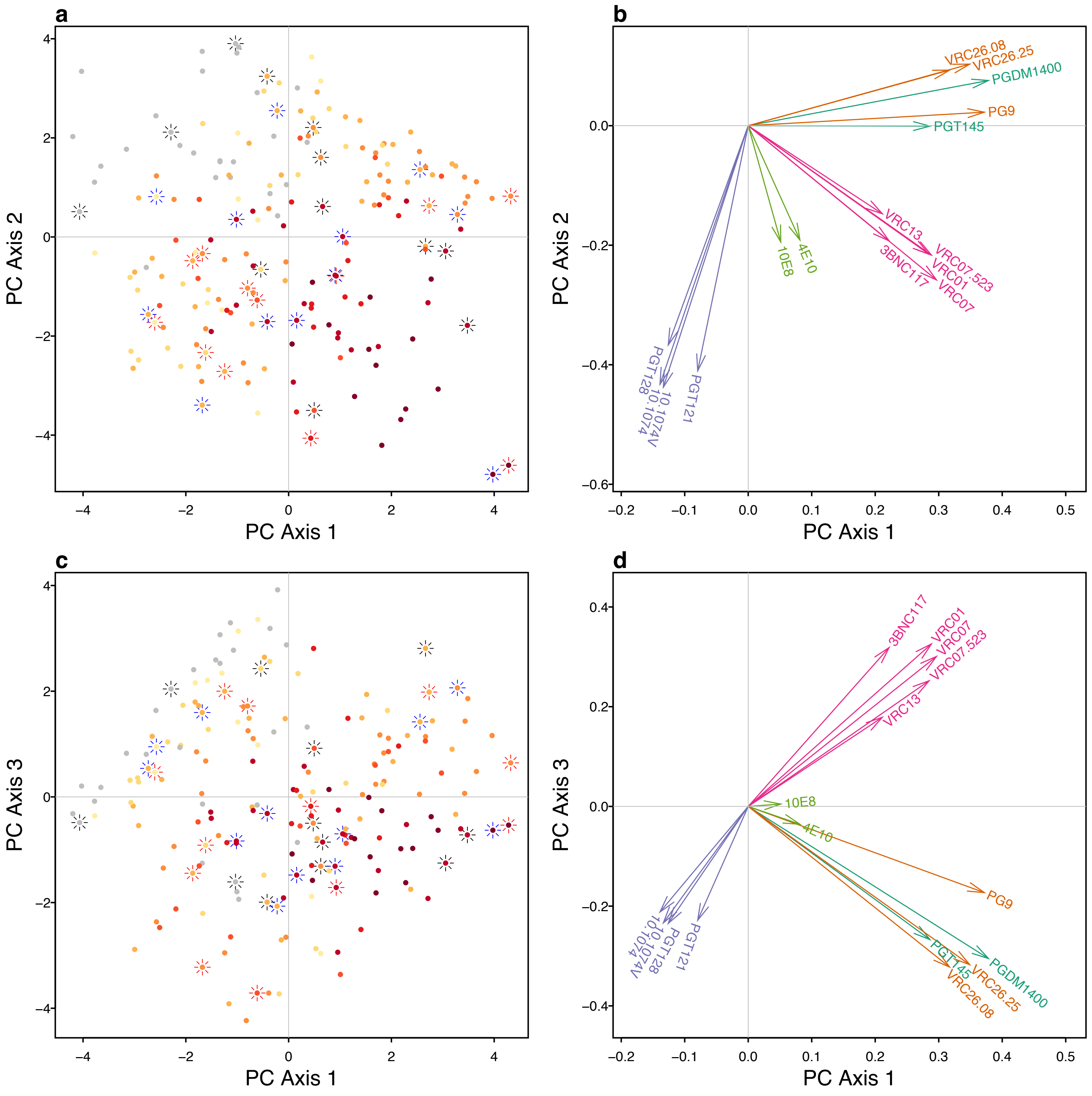
a
PC Axis 2
PC Axis 1
b
VRC26.08
VRC26.25
PGDM1400
PG9
PGT145
VRC13
VRC07.523
VRC01
VRC07
3BNC117
10E8
4E10
PGT128
10.1074V
10.1074
PGT121
PC Axis 2
PC Axis 1
c
PC Axis 3
PC Axis 1
d
3BNC117
VRC01
VRC07
VRC07.523
VRC13
10E8
4E10
PG9
PGDM1400
VRC26.25
VRC26.08
PGT145
PGT128
10.1074V
10.1074
PGT121
PC Axis 3
PC Axis 1

Figure S3

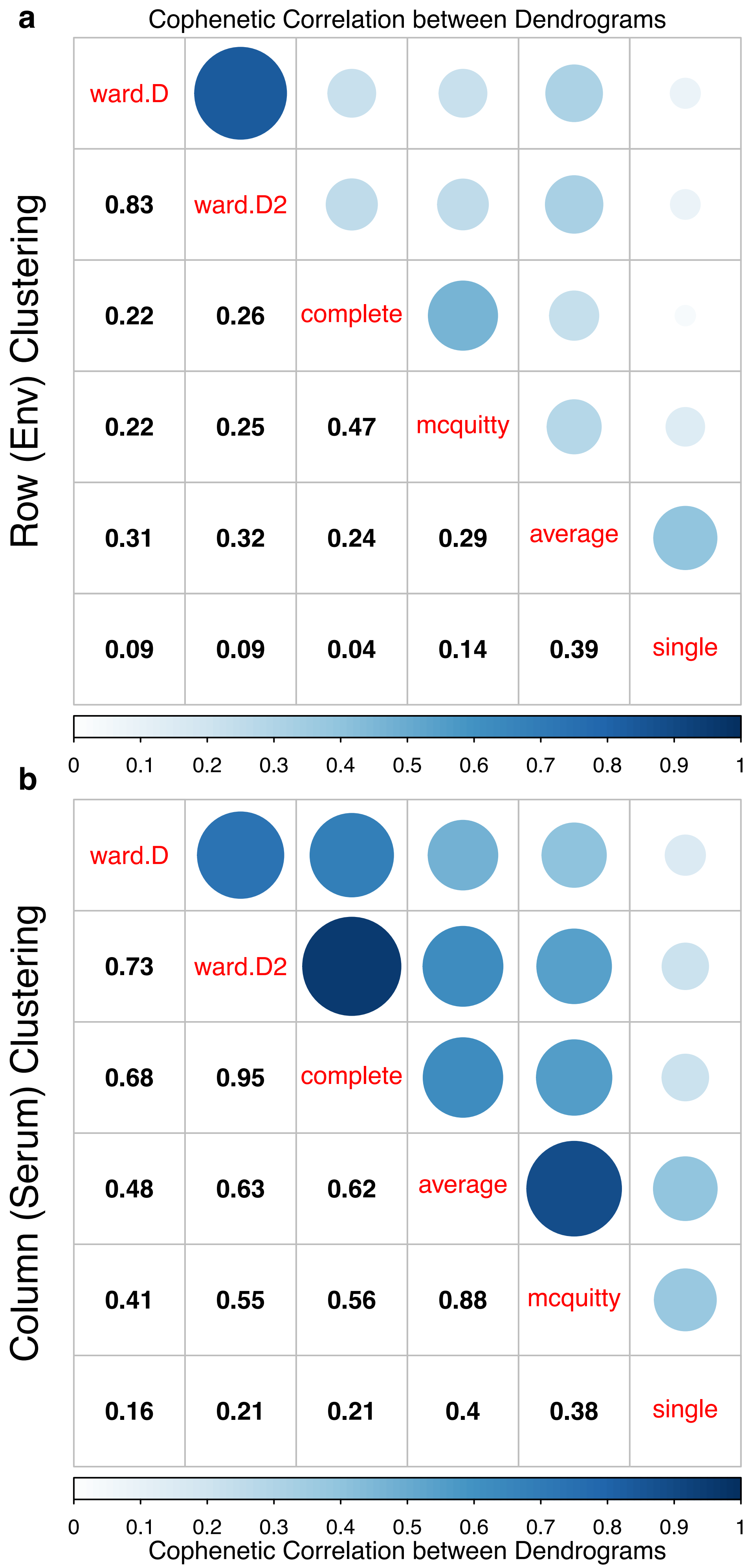

a
Cophenetic Correlation between Dendrograms
Row (Env) Clustering
ward.D
0.83
ward.D2
0.22
0.26
complete
0.22
0.25
0.47
mcquitty
0.31
0.32
0.24
0.29
average
0.09
0.09
0.04
0.14
0.39
single
0 0.1 0.2 0.3 0.4 0.5 0.6 0.7 0.8 0.9 1
b
Column (Serum) Clustering
ward.D
0.73
ward.D2
0.68
0.95
complete
0.48
0.63
0.62
average
0.41
0.55
0.56
0.88
mcquitty
0.16
0.21
0.21
0.4
0.38
single
0 0.1 0.2 0.3 0.4 0.5 0.6 0.7 0.8 0.9 1
Cophenetic Correlation between Dendrograms

Figure S4

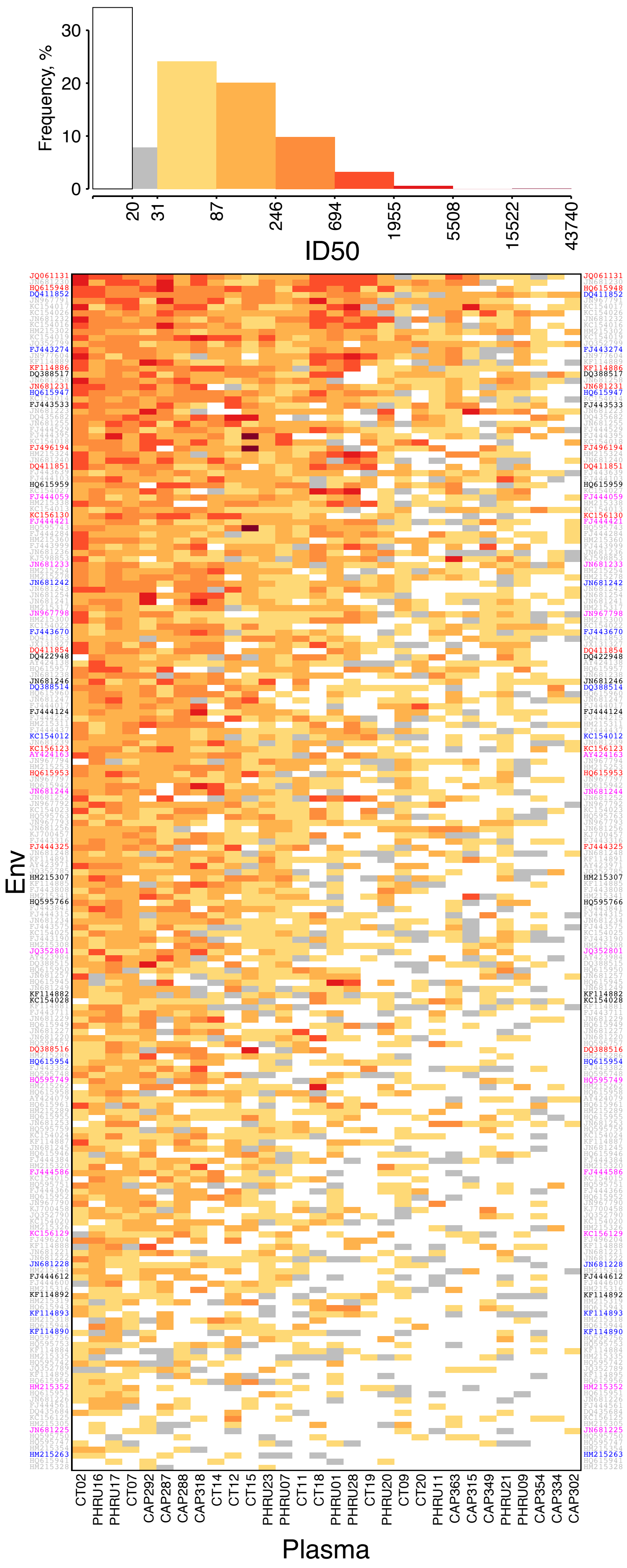
Frequency, %
ID50
Env
Plasma
CT02
PHRU16
PHRU17
CT07
CAP292
CAP287
CAP288
CAP318
CT14
CT12
CT15
PHRU23
PHRU07
CT11
CT18
PHRU01
PHRU28
CT19
PHRU20
CT09
CT20
PHRU11
CAP363
CAP315
CAP349
PHRU21
PHRU09
CAP354
CAP334
CAP302

Figure S5

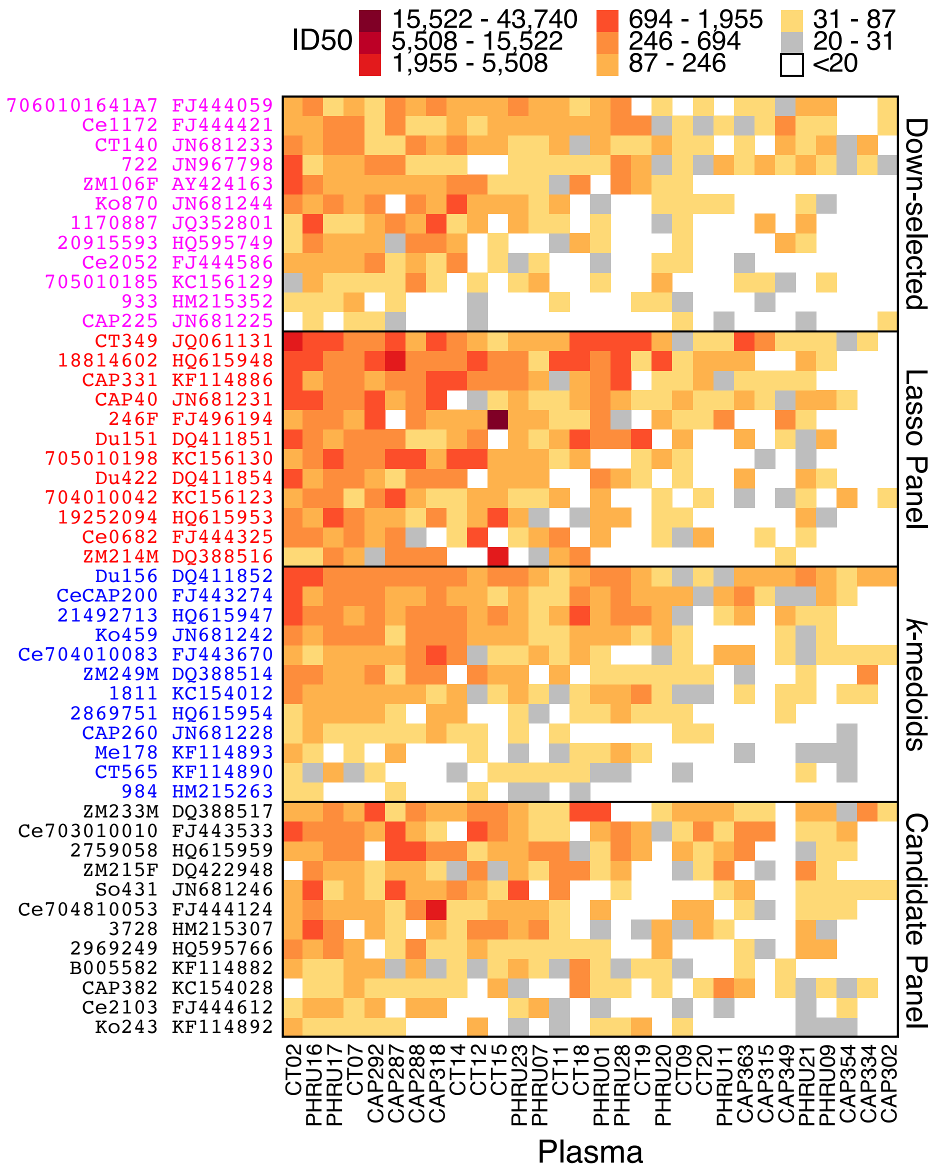
ID50
15,522 - 43,740
5,508 - 15,522
1,955 - 5,508
694 - 1,955
246 - 694
87 - 246
31 - 87
20 - 31
<20
7060101641A7 FJ444059
Ce1172 FJ444421
CT140 JN681233
722 JN967798
ZM106F AY424163
Ko870 JN681244
1170887 JQ352801
20915593 HQ595749
Ce2052 FJ444586
705010185 KC156129
933 HM215352
CAP225 JN681225
CT349 JQ061131
18814602 HQ615948
CAP331 KF114886
CAP40 JN681231
246F FJ496194
Du151 DQ411851
705010198 KC156130
Du422 DQ411854
704010042 KC156123
19252094 HQ615953
Ce0682 FJ444325
ZM214M DQ388516
Du156 DQ411852
CeCAP200 FJ443274
21492713 HQ615947
Ko459 JN681242
Ce704010083 FJ443670
ZM249M DQ388514
1811 KC154012
2869751 HQ615954
CAP260 JN681228
Me178 KF114893
CT565 KF114890
984 HM215263
ZM233M DQ388517
Ce703010010 FJ443533
2759058 HQ615959
ZM215F DQ422948
So431 JN681246
Ce704810053 FJ444124
3728 HM215307
2969249 HQ595766
B005582 KF114882
CAP382 KC154028
Ce2103 FJ444612
Ko243 KF114892
Down-selected
Lasso Panel
k-medoids
Candidate Panel
CT02
PHRU16
PHRU17
CT07
CAP292
CAP287
CAP288
CAP318
CT14
CT12
CT15
PHRU23
PHRU07
CT11
CT18
PHRU01
PHRU28
CT19
PHRU20
CT09
CT20
PHRU11
CAP363
CAP315
CAP349
PHRU21
PHRU09
CAP354
CAP334
CAP302
Plasma

Figure S6
